# Molecular hydrogen attenuates sepsis-induced cardiomyopathy in mice by promoting autophagy

**DOI:** 10.1186/s12871-024-02462-4

**Published:** 2024-02-23

**Authors:** Yan Cui, Yingning Li, Shuqi Meng, Yu Song, Keliang Xie

**Affiliations:** 1 Department of Pathogen Biology, School of Basic Medical Sciences, Tianjin Medical University, Tianjin, 300070 China; 2 Department of Anesthesiology, Tianjin Institute of Anesthesiology, Tianjin Medical University General Hospital, No. 154, Anshan Road, Heping District, Tianjin, 300052 China; 3 Department of Critical Care Medicine, Tianjin Medical University General Hospital, Tianjin, 300052 China

**Keywords:** Sepsis-induced cardiomyopathy, Hydrogen gas, Autophagy, Mitophagy

## Abstract

**Background:**

Approximately 40 to 60% of patients with sepsis develop sepsis-induced cardiomyopathy (SIC), which is associated with a substantial increase in mortality. We have found that molecular hydrogen (H_2_) inhalation improved the survival rate and cardiac injury in septic mice. However, the mechanism remains unclear. This study aimed to explore the regulatory mechanism by which hydrogen modulates autophagy and its role in hydrogen protection of SIC.

**Methods:**

Cecal ligation and puncture (CLP) was used to induce sepsis in adult C57BL/6J male mice. The mice were randomly divided into 4 groups: Sham, Sham + 2% hydrogen inhalation (H_2_), CLP, and CLP + H_2_ group. The 7-day survival rate was recorded. Myocardial pathological scores were calculated. Myocardial troponin I (cTnI) levels in serum were detected, and the levels of autophagy- and mitophagy-related proteins in myocardial tissue were measured. Another four groups of mice were also studied: CLP, CLP + Bafilomycin A1 (BafA1), CLP + H_2_, and CLP + H_2_ + BafA1 group. Mice in the BafA1 group received an intraperitoneal injection of the autophagy inhibitor BafA1 1 mg/kg 1 h after operation. The detection indicators remained the same as before.

**Results:**

The survival rate of septic mice treated with H_2_ was significantly improved, myocardial tissue inflammation was improved, serum cTnI level was decreased, autophagy flux was increased, and mitophagy protein content was decreased (*P* < 0.05). Compared to the CLP + H_2_ group, the CLP + H_2_ + BafA1 group showed a decrease in autophagy level and 7-day survival rate, an increase in myocardial tissue injury and cTnI level, which reversed the protective effect of hydrogen (*P* < 0.05).

**Conclusion:**

Hydrogen exerts protective effect against SIC, which may be achieved through the promotion of autophagy and mitophagy.

**Supplementary Information:**

The online version contains supplementary material available at 10.1186/s12871-024-02462-4.

## Introduction

Sepsis-induced cardiomyopathy (SIC) is a significant contributor to the poor prognosis of sepsis patients. It has been reported that SIC develops in approximately 40 to 60% of sepsis patients. The mortality rate is around 20% for sepsis patients without SIC, whereas it goes up to 70% for those with SIC [1]. Currently, there is no optimal treatment available for SIC, and understanding its pathogenesis and developing effective treatment strategies remain key challenges in the field of critical care medicine. Most current research focuses on the role of inflammatory mechanisms in sepsis [2, 3]. Autophagy is a lysosome-dependent self-degradation process that plays a crucial role in cell survival. Mitophagy is a form of autophagy, which is a process of selective removal of damaged mitochondria [4]. Autophagy dysfunction has a substantial impact on the pathogenesis of various human diseases, including metabolic diseases, cardiovascular diseases, cancer, sepsis, infectious diseases, and so on [5–7]. Several studies have demonstrated the significant role of abnormal autophagy regulation in SIC [8, 9].

In the last decade, molecular hydrogen (H_2_) has emerged as a promising medical gas with powerful antioxidant and anti-inflammatory properties [10, 11]. Numerous studies have demonstrated the beneficial therapeutic effects of H_2_ on various diseases, including cardiovascular disease, respiratory disease, infection, and neurodegenerative diseases. However, the exact mechanisms underlying these effects are yet to be fully clarified [12–14]. In the case of SIC, our previous research has shown that hydrogen therapy improves the survival rate of sepsis mice and restores cardiac function [11]. Furthermore, several studies have indicated a link between SIC and autophagy. Our previous investigations have revealed that hydrogen can modulate multiple signaling pathways involved in autophagy, endoplasmic reticulum stress, and cellular energy metabolism [15–17]. Nevertheless, it remains unclear whether the protective effects of hydrogen on SIC are mediated through autophagy. Therefore, we conducted a study using a mouse model of SIC induced by cecal ligation puncture (CLP) to explore the role of autophagy in the amelioration of SIC by hydrogen therapy.

## Materials and methods

### Animals

The Laboratory Animal Center of the Chinese Academy of Medical Sciences in Beijing, China, provided us with healthy clean grade male C57BL/6J mice aged 6–8 weeks, weighing 20–25 g. The mice acclimated to the environment for 1 day before the experiment. All the mice were housed at a temperature of 20 to 22 ℃ and a humidity of 60 − 80%, and kept on a 12 h/12 h light/dark cycle, and food and water were available at any time. All the experimental mice were handled humanely. All animal studies were conducted according to the ARRIVE guidelines and approved by the Experimental Animal Ethics Committee of Tianjin Medical University.

### Study protocol

This study consists of two experiments.

Experiment 1: Hydrogen promotes autophagy to alleviate cardiomyopathy in septic mice.

Mice were divided into 4 groups by random number method (*n* = 32/group): Sham, Sham treated with H_2_ (Sham + H_2_), CLP, and CLP treated with H_2_ (CLP + H_2_). All mice were anesthetized with 2% isoflurane in pure oxygen before CLP procedure. Mice in Sham + H_2_ group and CLP + H_2_ group inhaled 2% hydrogen for 1 h at 1 h and 6 h after the operation, meanwhile mice in Sham + H_2_ group and CLP group inhaled air. Twenty mice were randomly selected from each group to observe the survival rate until 7 days after operation. Six mice in each group were randomly selected and sacrificed 24 h after operation, and their hearts were taken for paraffin section and Hematoxylin-eosin (HE) staining to observe the myocardial histopathological changes. 24 h after operation, 6 mice were randomly selected from each group to test Murine Sepsis Score(MSS) [18]. After the MSS evaluation, isoflurane anesthesia was performed to collect blood samples, and serum cTnI concentration was detected by ELISA. Then hearts of these mice were taken and western blotting was used to detect the expression levels of autophagy related proteins. All experimenters were blinded to the group assignment.

Experiment 2: Autophagy inhibitor BafA1 reverses the protective effect of hydrogen on SIC.

Mice were divided into 4 groups by random number method (*n* = 32/group): CLP, CLP treated with BafA1 (CLP + BafA1), CLP treated with H_2_ (CLP + H_2_), and CLP treated with BafA1 and H_2_ (CLP + H_2_ + BafA1). Mice were anesthetized with 2% isoflurane in pure oxygen before CLP procedure. Mice in CLP + H_2_ group and CLP + H_2_ + BafA1 group inhaled 2% hydrogen for 1 h at 1 h and 6 h after the operation, meanwhile mice in CLP group and CLP + BafA1 group inhaled air. The autophagy inhibitor Bafilomycin A1 (MCE Company, China) was allocated as a solution with a concentration of 0.25 mg/ml. Mice in CLP + BafA1 group and CLP + H_2_ + BafA1 group were intraperitoneally injected with BafA1 1 mg/kg 1 h after operation. Meanwhile mice in CLP group and CLP + H_2_ group were injected with equal dose of normal saline. Twenty mice were randomly selected from each group to observe the survival rate until 7 days after operation. Six mice in each group were randomly selected and sacrificed 24 h after operation, and their hearts were taken for paraffin section and haematoxylin-eosin (HE) staining to observe the myocardial histopathological changes. 24 h after operation, 6 mice were randomly selected from each group to test Murine Sepsis Score(MSS). After the MSS evaluation, isoflurane anesthesia was performed to collect blood samples, and serum cTnI concentration was detected by ELISA. Then hearts of these mice were taken and western blotting was used to detect the expression levels of autophagy related proteins. All experimenters were blinded to the group assignment.

### SIC model

SIC model in mice was induced by CLP. Mice were placed supine on a clean operating surface after 2% isoflurane anesthesia. Abdominal hair was removed and the skin was disinfected with iodor. A 1 cm longitudinal midline incision was made. The cecum was explored with smooth forceps. Mouse model with moderate sepsis: 60% of the cecum was ligated with surgical sutures, followed by two punctures with a 22-gauge needle at the midpoint of the caudal end of cecum ligation. After manually extruding part of the intestinal content, the cecum was returned to the abdominal cavity and the abdominal tissues were sutured layer by layer with a 3–0 surgical suture. Cecal exploration operation was performed in Sham group and Sham + H_2_ group, but no cecal ligation or perforation. The mice were resuscitated after subcutaneous injection of 0.9% sodium chloride solution 1 ml/20 g liquid into the neck, then placed on warm blankets until they woke up and returned to their cages. The antibiotic meropenem 20 mg/kg was injected intraperitoneally 6 h after operation.

### 2% H_2_ treatment

Hydrogen gas was prepared using a hydrogen generator (model AMS-H-03; Asclepius Meditec Co., Ltd., Shanghai, China). The mice were put into a sealed plexiglass chamber with an air outlet and an air inlet, and the hydrogen concentration was adjusted to 2% by mixing hydrogen with air. PG610 hydrogen concentration detector (Yingte electrical equipment Co., LTD, Henan, China) was used to measure the hydrogen concentration at 2%, and ST8100A oxygen detector (Smart Sensor, Kuangdong, China) was used at the same time, the oxygen concentration was 20.9%.

### Myocardial pathology assessment

Myocardial pathology was assessed by the HE staining method. Under deep anesthesia, the exposed mouse hearts were first injected with normal saline and then 4% paraformaldehyde. Mice heart tissues were taken, fixed in 4% paraformaldehyde solution, embedded in paraffin, and cut into 5 μm thick sections. Samples were dewaxed before HE staining and then stained. Based on previous studies [19], two pathologists, who were unaware of the experiment, scored myocardial pathological changes under the microscope BX51 (Olympus Corporation, Tokyo, Japan) based on the percentage of cells that showed myofibrillar loss or cytoplasmic vacuolation: 0 = no damage, 1 = < 5%, 2 = 5–30%, 3 = > 30%.

### Enzyme-linked immunosorbent assay (ELISA)

24 h after operation, 6 mice in each group were randomly collected and blood samples were collected under sevoflurane anesthesia. These blood samples were left at room temperature for 30 min, centrifuged at 3000 RPM for 10 min, and then the supernatant was collected. According to the manufacturer’s instructions, the serum cTnI concentration was detected using mouse ELISA kit (Elabscience Biotechnology Co., LTD., Wuhan, China).

### Western blotting analysis

After blood collection, mice were sacrificed under deep anesthesia, the whole heart was taken, and the atrium was cut off. The tissues were weighed, RIPA lysis buffer and protease inhibitor were added, ground on ice and crushed by an ultrasonic cell crusher (Xinzhi Biotechnology Co., Ltd., Ningbo, China), then supernatant was obtained. The protein concentration was determined using the BCA Protein Assay Kit (Biosharp Co., Ltd.). Loading buffer (Biosharp Co., Ltd.) was added, boiled for 10 min and cooled to room temperature. Electrophoresis was performed using 8-16% and 10% gel, and transferred to polyvinylidene fluoride (PVDF) membranes (0.45 μm, Millipore Co., Ltd.). The membranes were then blocked by incubation in 10% nonfat milk at room temperature for 1.5 h, then incubated with primary antibodies at 4 ℃ overnight and incubated with secondary antibodies at room temperature for 1 h. The protein bands were then visualised by ECL development and exposure (ProteinSample, USA). The primary antibodies used were anti-LC3B (1:2000, Abcam, USA), anti-P62 (1:500, Abcam, USA), anti-PINK1 (1:200, Abcam, USA), anti-Parkin (1:2000, Abcam, USA), and anti-GAPDH (1:5000, Affinity Biosciences, USA). The secondary antibodies used were goat anti-rabbit (1:5000, Affinity Biosciences, USA) and goat anti-mouse (1:5000, Aorui Dongyuan Biotechnology Co., LTD., Wuxi, China). The protein bands exposure instrument (Proteinsimple, FluorChem, USA) could only expose the protein bands, and could not take photos and synthesize the whole protein membrane in advance. The protein bands provided in this paper were all cropped protein bands. The gray values of the bands were quantified by ImageJ software, and the expression levels of target proteins were standardised with GAPDH level.

### Statistical analysis

SPSS26.0 software was used for analysis. The survival rate of each group was expressed as percentage (%), and the measurement data of normal distribution were expressed as mean ± standard deviation (x ± s). One-way ANOVA was used for comparison between groups, and Fisher exact probability method was used for comparison of survival rates. *P* < 0.05 was considered statistically significant.

## Results

### H_2_ inhalation improves the survival rates of septic mice and murine Sepsis score

**Fig. 1:**
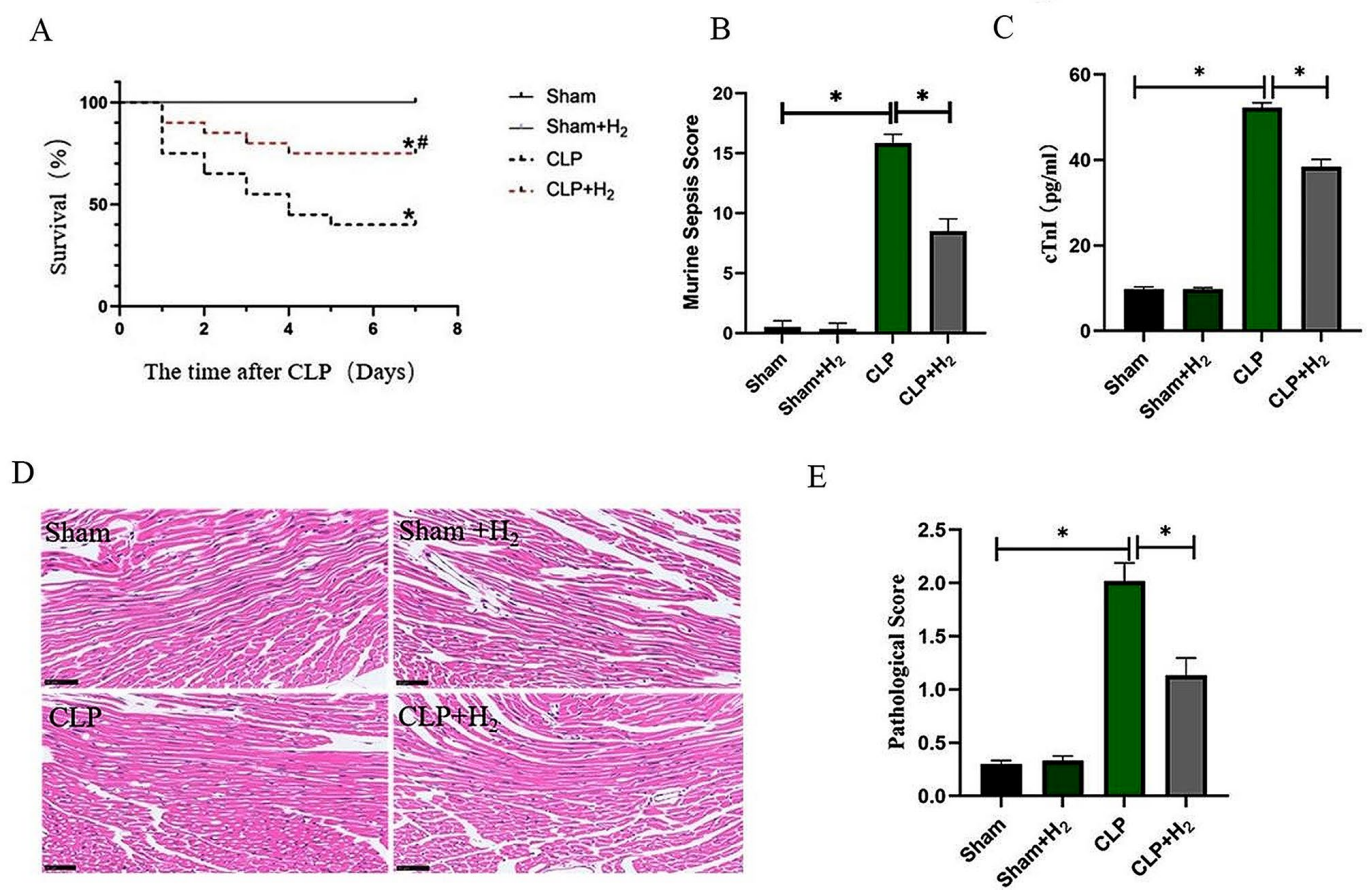
2% H_2_ inhalation elevated the 7-day survival rate of the septic mice and alleviated myocardial injury. (**A**) The 7-day survival rate (*n* = 20, log-rank test), *P < 0.05 vs. Sham group, ^*#*^*P* < 0.05 vs. CLP group. (**B**) Murine Sepsis Score (*n* = 6), (**C**) Serum cTnI level (*n* = 6), (**D**) HE staining of mouse myocardial tissue (×40) (*n* =6), and (**E**) the myocardial pathological score (*n* = 6) were measured in each group. *P < 0.05

The 7-day survival rate of mice is shown in Table 1, and the 7-day survival curve is shown in Fig. 1. As shown in Fig. 1A, the 7-day survival rates of both the Sham group and Sham + H_2_ group were both 100%, with no statistical significance (*P >* 0.05). However, compared to the Sham group, the 7-day survival rates for the CLP group and CLP + H_2_ group were 40% (*P* < 0.05) and 75% (*P* < 0.05), respectively. Mice in the CLP group showed obvious mental fatigue, drowsiness, less movement, purulent secretion in the corner of the eyes, and coarse and dull hair. Therefore, we tested the murine sepsis score (MSS). As shown in Fig. 1B, compared with the Sham group, the MSS of the CLP group was significantly increased (*P* < 0.05), and the MSS was obviously decreased after H_2_ treatment (*P* < 0.05).

**Table 1:** The effect of H_2_ on the survival rate of mice (%)

	1d	2d	3d	4d	5d	6d	7d
Sham	100	100	100	100	100	100	100
Sham + H_2_	100	100	100	100	100	100	100
CLP	75	65	55	45	40	40	40^*^
CLP + H_2_	90	85	80	75	75	75	75^#^

^*^P< 0.05 versus the Sham group, ^#^*P* < 0.05 versus the CLP group. Data are expressed as mean ± SD (*n* = 20)

### H_2_ inhalation alleviates pathological changes and serum markers of SIC

The serum cTnI levels in each group were detected 24 h after operation. Compared to the Sham group, cTnI levels were elevated 24 h after the operation in both the CLP group and CLP + H_2_ group (*P* < 0.05). Compared to the CLP group, cTnI level in the CLP + H_2_ group was decreased (*P* < 0.05) (Fig. 1C).

Twenty-four hours after the operation, myocardial fibers in the Sham group and Sham + H_2_ group were observed under a high-power light microscope, revealing closely arranged and well-structured fibers. Compared with the Sham group, the myocardial fibers in the CLP group exhibited evident edema, disordered, broken and vacuolated, accompanied by infiltration of inflammatory cells and overflow of red blood cells. As shown in Fig. 1C, compared with the CLP group, the arrangement of myocardial fibers in the CLP + H_2_ group appeared more regular, with slight cellular edema, reduced fiber rupture and cytoplasmic vacuolation, and a small amount of inflammatory cell infiltration (Fig. 1D). Furthermore, following the aforementioned methodology, the myocardial pathological scores in the CLP + H_2_ group were significantly higher than those in the CLP group (*P* < 0.05) (Fig. 1E).

### H_2_ inhalation affects autophagy in septic mice

**Fig. 2:**
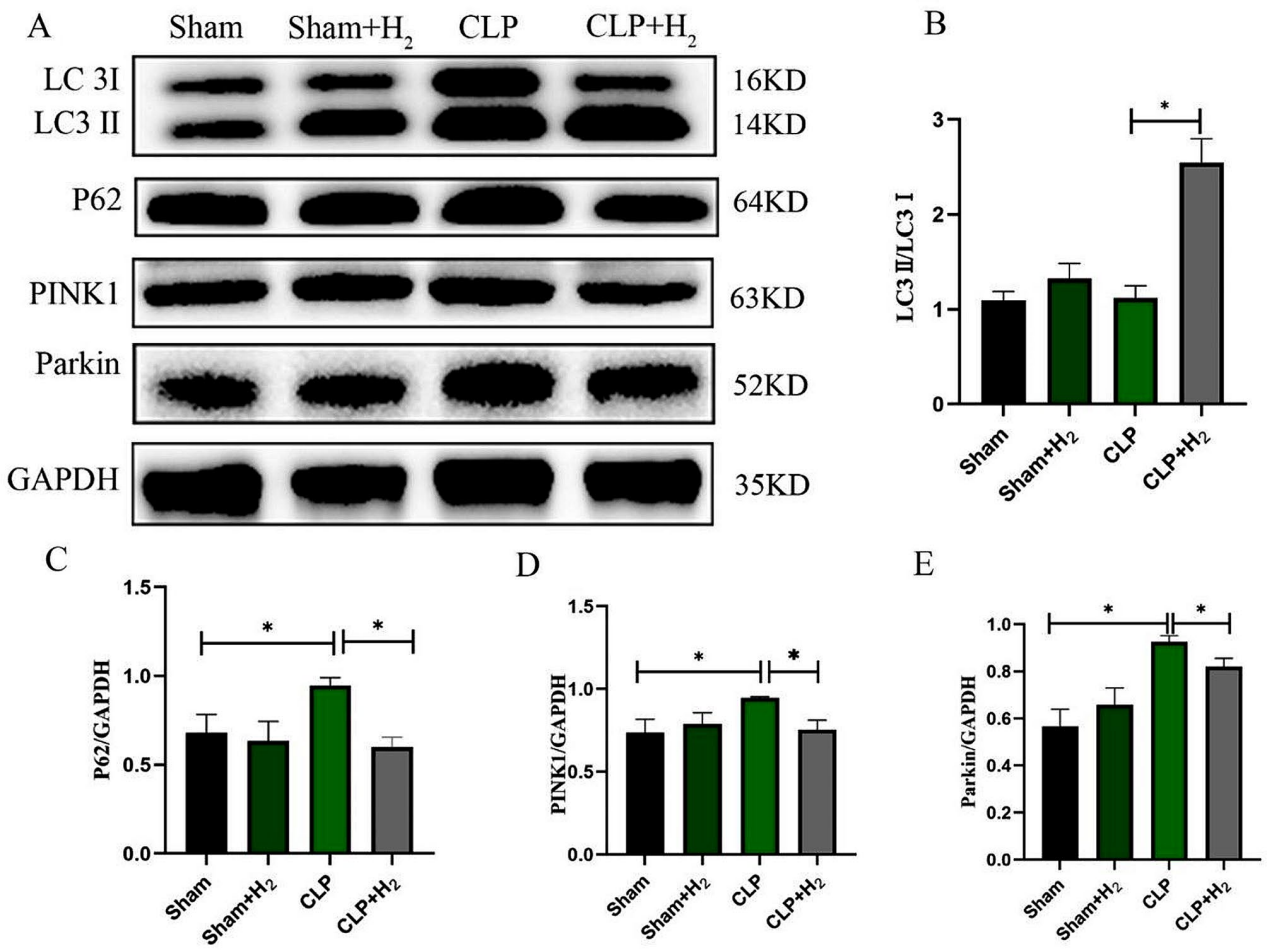
Effects of 2% hydrogen on myocardial autophagy and mitophagy in septic mice. (**A**-**E**) Expression levels of autophagy- and mitophagy-related proteins (*n* = 6). The data are expressed as mean ± SD. *P < 0.05

Western Blot was used to detect the expression levels of autophagy-related proteins. We found that compared to the Sham group, there was no significant change in the LC3II/LC3I ratio within the CLP group. However, the levels of P62 protein and phosphatase and tensin homolog (PTEN) induced putative kinase 1 (PINK1)/Parkin were both significant increased (*P* < 0.05). On the other hand, in the CLP + H_2_ group, the LC3II/LC3I ratio was increased, while the levels of P62 and PINK1/Parkin were both decreased when compared to the CLP group, and all of these differences were statistically significant (*P* < 0.05) (Fig. 2A-E).

### BafA1 significantly inhibits autophagy in septic mice

**Fig. 3:**
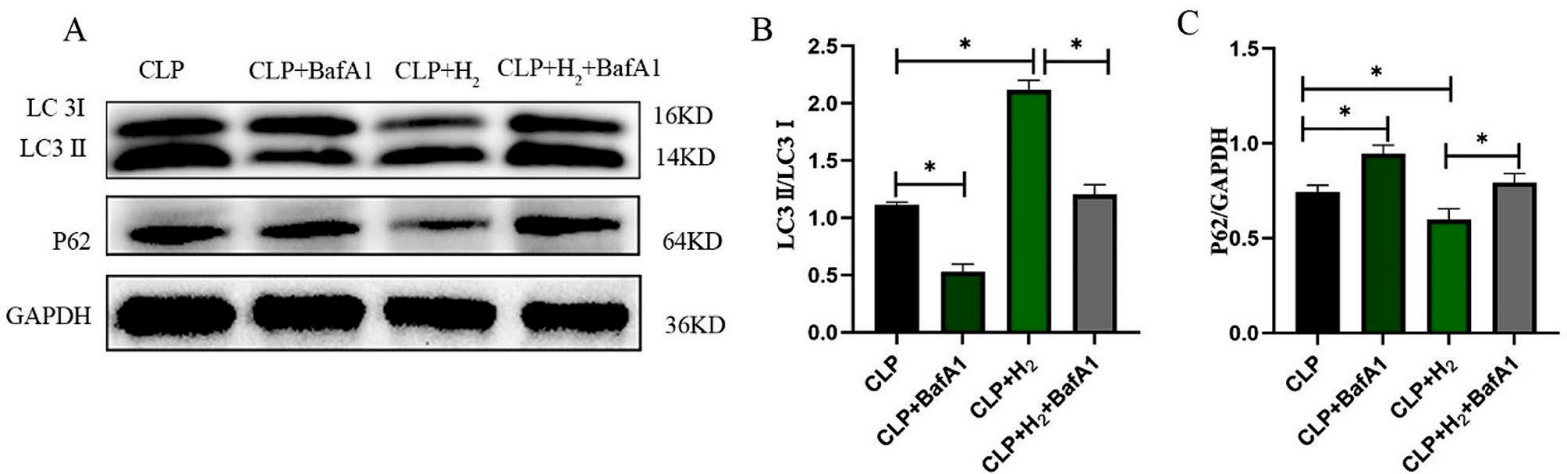
BafA1 significantly inhibits myocardial autophagy in septic mice. (**A**-**C**) Expression levels of autophagy-related proteins (*n* = 6). The data are expressed as mean ± SD. *P < 0.05

The autophagy levels in myocardial tissue were detected 24 h after operation by western blotting. As shown in Fig. 3A-C, we observed that, in comparison to the CLP group, there was a decrease in the LC3II/LC3I ratio and an increase in P62 level in the CLP + BafA1 group (*P* < 0.05). Furthermore, compared to the CLP + H_2_ group, there was a decrease in the LC3II/LC3I ratio and an increase in P62 level in the CLP + H_2_ + BafA1 group (*P* < 0.05).

### BafA1 reduces the survival rate of septic mice and murine sepsis score

**Fig. 4:**
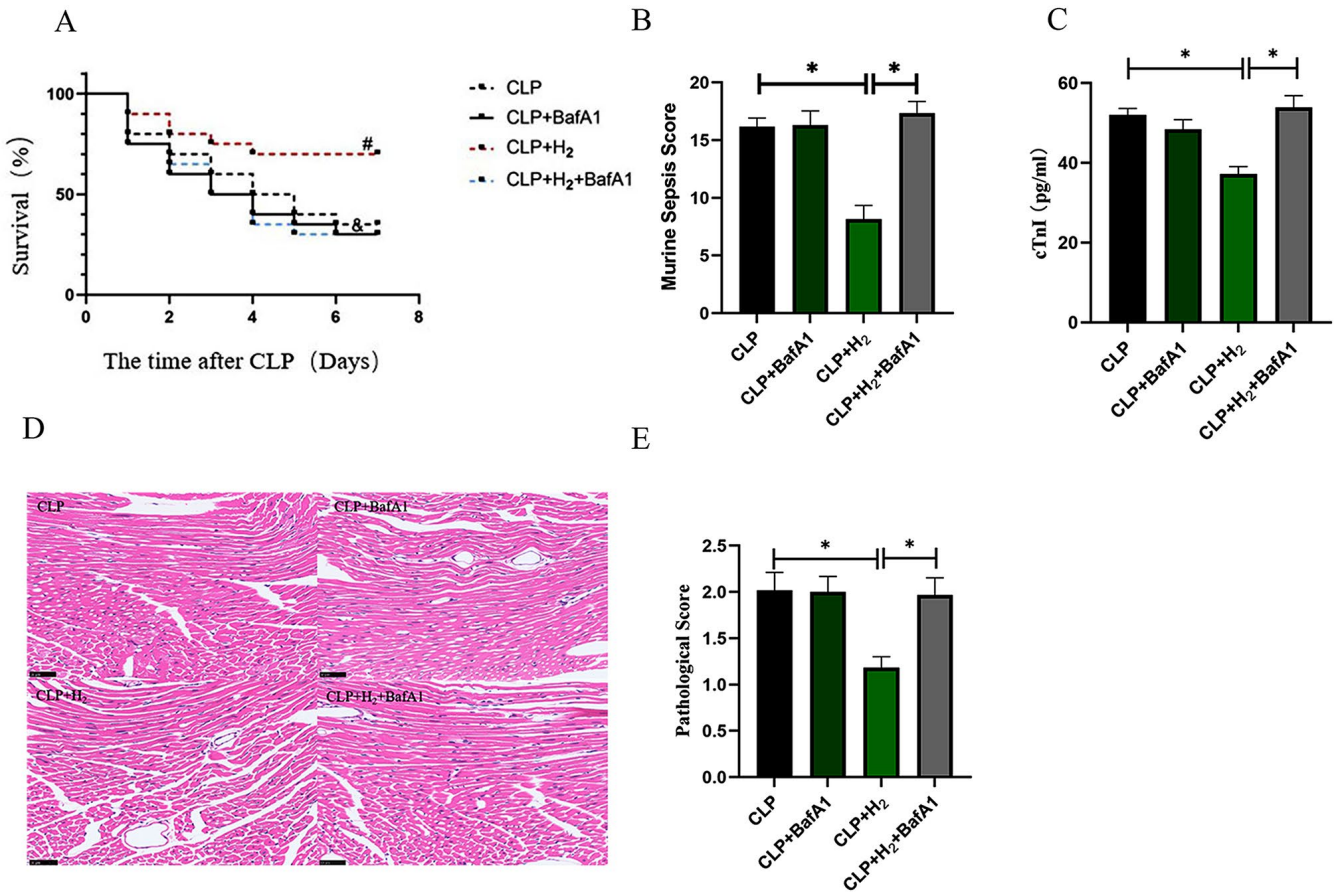
Effects of BafA1 on the 7-day survival rate and myocardial injury in septic mice. (**A**) The 7-day survival rate (*n* = 20, log-rank test), ^*#*^*P* < 0.05 vs. CLP group, ^*&*^*P* < 0.05 vs. CLP + H_2_ group. (**B**) Murine Sepsis Score (*n* = 6), (**C**) Serum cTnI level (*n* = 6), (**D**) HE staining of mouse myocardial tissue (×40) (*n* = 6), and (**E**) the myocardial pathological score (*n* = 6) were measured in each group. *P < 0.05

As shown in Fig. 4A, the 7-day survival rates in the CLP, CLP + BafA1, CLP + H_2_, and CLP + H_2_ + BafA1 groups were 35% (7/20), 30% (6/20), 70% (14/20), and 30% (6/20), respectively. There was no significant difference between the CLP group and CLP + BafA1 group (*P >* 0.05). Compared with CLP group, the 7-day survival rate in the CLP + H_2_ group was improved (*P* < 0.05). Compared with CLP + H_2_ group, the 7-day survival rate in the CLP + H_2_ + BafA1 group was lower (*P* < 0.05). The 7-day survival rate of mice is shown in Table 2. In addition, compared with the CLP group, MSS was significantly improved after H_2_ inhalation (*P* < 0.05). However, the MSS of the CLP + H_2_ group increased again after BafA1 used (*P* < 0.05), which were shown in Fig. 4B.

**Table 2:** The effect of BafA1 on the survival rate of mice (%)

	1d	2d	3d	4d	5d	6d	7d
CLP	75	65	55	45	40	35	35
CLP + BafA1	75	60	50	40	35	30	30
CLP + H_2_	90	85	80	75	70	70	70^#^
CLP + H_2_ + BafA1	80	65	50	35	30	30	30^&^

^#^*P* < 0.05 versus the CLP group, ^&^*P* < 0.05 versus the CLP + H_2_ + BafA1 group. Data are expressed as mean ± SD (*n* = 20)

### BafA1 increases pathological changes and elevates serum markers of SIC

Myocardial tissue was observed by HE staining under a high-power light microscope. 24 h after operation, the CLP + H_2_ + BafA1 group exhibited more obvious myocardial tissue cell edema, an increased ratio of cytoplasmic vacuolation, and elevated interstitial inflammatory cell infiltration when compared to the CLP + H_2_ group. This indicated aggravated inflammation and evident injury in the myocardium. Moreover, both the pathological score and cTnI content were significantly increased (*P*<0.05) (Fig. 4C-E).

## Discussion

In this study, we found that inhalation of 2% hydrogen can improve the survival rate of sepsis mice, alleviate myocardial pathological injury, and reduce serum marker of SIC. This beneficial effect was achieved by promoting both autophagy and mitophagy in cardiomyocyte. Notably, when an autophagy inhibitor was added, it reversed the protective effects of hydrogen in SIC, underscoring the important role of autophagy in mediating these effects.

Cardiac dysfunction caused by sepsis is a crucial part of multiple organ failure in sepsis, contributing to a relatively high mortality rate [20]. The 7-day mortality rate of the CLP mouse model established in this study was 60%, which is consistent with previous studies [21]. Hydrogen, as a novel medical gas molecule, has been confirmed to possess anti-inflammatory and antioxidant properties. Numerous studies have demonstrated its effectiveness in mitigating functional damage in organs such as the brain, lungs, and intestines during sepsis [22–24]. With reference to literature, the concentration of troponin I (cTnI) in serum was employed as a marker to evaluate myocardial injury in sepsis [25]. The findings of this study revealed that inhalation of 2% hydrogen at 1 and 6 h respectively after CLP surgery significantly improved the 7-day survival rate of mice, alleviated myocardial tissue edema and inflammatory cell infiltration, and decreased the serum marker of SIC. The above results confirmed the therapeutic potential of hydrogen in alleviating myocardial injury, which was consistent with previous studies [11, 26].

The pathogenesis of SIC is complex, mainly involving inflammatory mediators disorders, mitochondrial dysfunction, autophagy dysregulation, oxidative stress, calcium regulation disorders, endothelial dysfunction, etc. [1, 27, 28]. . Numerous studies have recognized the critical role of autophagy regulation in SIC [8, 27]. In sepsis, there is an upregulation of autophagy activity in myocardial tissue, aiding in the clearance of pathogens and damaged organelles [29]. A recent study showed that blocking the mTOR pathway accelerated autophagy, leading to a protective effect against CLP-induced SIC [30]. Another study revealed that enhancing AMPK expression improved myocardial systolic function in septic mice by increasing autophagy activity [31]. Mitophagy, a specialized type of autophagy, selectively eliminates excess or damaged mitochondria to maintain mitochondrial quantity and function stability [32]. Given that cardiomyocytes, with their high energy demands, heavily rely on ATP generated by mitochondria for normal function, impaired mitochondrial function poses a fatal threat to the septic heart, and mitophagy serves as an essential self-repair pathway [33]. Yue Wang et al. found that down-regulating myocardial mitochondrial autophagy in SIC mice led to reduced ATP production levels in cardiomyocytes and increase myocardial damage [34].

In this study, with reference to literature, autophagy activity in septic myocardial tissue was assessed by measuring the levels of LC3-II/I and P62 protein, while mitochondrial autophagy activity was evaluated by detecting PINK1/Parkin levels. LC3 (microtubule-associated protein 1 light chain 3, LC3) is a membrane protein located on the autophagosome. LC3-I is synthesized immediately after autophagy initiation, and it subsequently transforms into mature LC3-II through binding to autophagy proteins. Mature LC3-II is located on the fully formed autophagosome. Therefore, LC3-II/I level reflects the progression of autophagy and serves as a reliable indicator of autophagy activity. An increase in just LC3-II content cannot solely represent the upregulation of autophagy activity, which may be due to the inability of autophagosome to fuse with lysosome and undergo degradation, resulting in a state of accumulation [35]. Another commonly used marker to assess autophagy activity is the P62 protein. P62 is degraded via autophagy, and its content exhibits a negative correlation with autophagy activity, that is, P62 accumulates upon autophagy inhibition and decreases upon autophagy induction [36]. Mitophagy mainly encompasses three pathways: PINK1/Parkin, BNIP3, and FUNDC1 [37]. The PINK1/Parkin pathway plays a central role in the ubiquitination process, becoming activated when changes occur in mitochondrial membrane potential following mitochondrial damage. Upon activation, PINK1/Parkin is transferred to the outer membrane of mitochondria, where it is recognized by autophagosomes and subsequently transported to lysosomes through phagocytosis [38]. Our findings demonstrated that in the myocardial tissue of the CLP group, there was no statistically significant difference in the ratio of LC3-II/I compared to the Sham group. However, the content of P62 protein showed a significant increase, indicating a blockade in autophagy flux. Both PINK1 and Parkin expressions were elevated, suggesting increased mitophagy or protein accumulation caused by the obstruction of autophagy. In the CLP + H_2_ group, compared to the CLP group, the ratio of LC3-II/I was significantly increased, while P62 content decreased, indicating that hydrogen could promote autophagy. However, both PINK1 and Parkin levels decreased, suggesting that the upregulation of mitophagy protein at 24 h after CLP might be caused by protein accumulation due to the restriction of mitophagy, and hydrogen promoted the sustained progression of mitophagy. These results suggest that hydrogen may alleviate SIC by enhancing autophagy and mitophagy processes.

Autophagy is a complex and dynamic multi-step process. During different stages of sepsis, autophagy activity changes constantly [39]. Studies have demonstrated that the application of rapamycin can induce complete myocardial autophagy process in sepsis, leading to reduced myocardial inflammatory response and improved cardiac function. Conversely, the use of the autophagy inhibitor BafA1 did not yield any benefits [40]. In our study, we observed that hydrogen therapy can significantly increase the LC3-II/LC3-I ratio and decrease the level of autophagy substrate P62. Hence, we hypothesized that hydrogen may promote the continuation of late-stage autophagy, that is, the binding and degradation process of autophagosomes and lysosomes. To validate this hypothesis, we used the late-stage autophagy inhibitor BafA1, which inhibits autophagolysosomes acidification by blocking V-ATPase [41]. The results showed that BafA1 treatment significantly inhibited autophagy, as evidenced by a decrease in the LC3-II/LC3-I ratio and an increase in P62 expression. Furthermore, when autophagy was blocked, myocardial damage was also aggravated. These findings suggested that BafA1 reversed the myocardial protective effect of hydrogen by blocking autophagy.

Hydrogen, as a gas with anti-inflammatory and antioxidant properties, has the potential to alleviate the systemic inflammatory response in sepsis, reduce tissue and blood levels of inflammatory factors, and modulate macrophage function [12, 14]. Additionally, hydrogen can interact with oxygen free radicals, thereby reducing oxidative stress in the body [10]. Consequently, we hypothesized that hydrogen could mitigate the dysregulation of autophagy in septic myocardial cells by alleviating excessive inflammatory responses and oxidative stress. Previous studies have demonstrated that hydrogen can protect against sepsis-induced damage in the brain, lungs, nerves, and other tissues by regulating autophagy activity. However, no studies have explored whether hydrogen can safeguard the myocardium from sepsis-induced damage through autophagy.

There are some limitations in our study. We have only confirmed the association between the protective effect of hydrogen on SIC and its promotion of autophagy and mitophagy. However, we have to identify the specific signaling pathway through which hydrogen regulates autophagy to exert its protective effects on SIC. Future studies should focus on elucidating the precise target of hydrogen in autophagy initiation and autophagosomal formation, so as to lay an experimental foundation for uncovering novel mechanisms for SIC therapy.

## Conclusion

In conclusion, our study demonstrates that hydrogen can improve the 7-day survival rate of septic mice and ameliorate myocardial injury by promoting autophagy and mitophagy. These findings offer a novel therapeutic approach for the treatment of SIC.

### Electronic supplementary material

Below is the link to the electronic supplementary material.

Supplementary Material 1

Supplementary Material 2

Supplementary Material 3

Supplementary Material 4

Supplementary Material 5

Supplementary Material 6

Supplementary Material 7

Supplementary Material 8

Supplementary Material 9

Supplementary Material 10

## Data Availability

The datasets used and/or analysed during the current study available from the corresponding author on reasonable request.

## Abbreviations

SIC: sepsis-induced cardiomyopathy
H_2_: molecular hydrogen
CLP: cecal ligation and puncture
HE: hematorylin-eosin
ELISA: enzyme-linked immunosorbent assay
