# Supplementary figures and images for: Molecular hydrogen attenuates sepsis-induced cardiomyopathy in mice by promoting autophagy
Source: BMC Anesthesiol. 2024 Feb 23;24:72. doi: 10.1186/s12871-024-02462-4 (PMC10885652; doi:10.1186/s12871-024-02462-4)

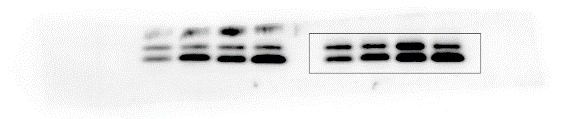
**LC3Ⅰ & LC3Ⅱ**


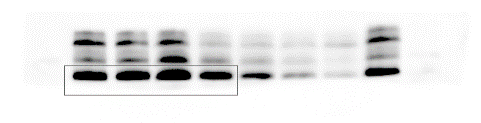
 **P62**

**
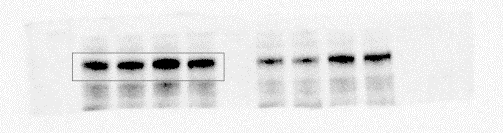
 Parkin**

**
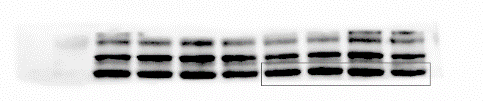
 PINK**

**
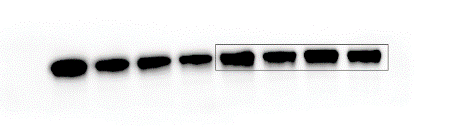
 GAPDH**

Supplementary figure 2A. Western Blot full-length blots.

Supplement: Supplementary file 6 — Supplementary Material 6 [file 12871_2024_2462_MOESM6_ESM.docx]

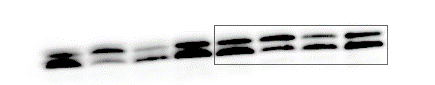
 **LC3Ⅰ & LC3Ⅱ**

**
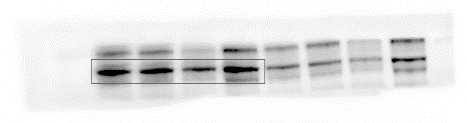
 P62**

**
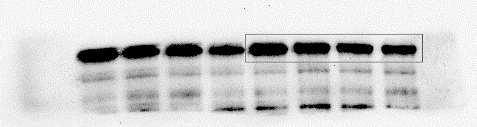
 GAPDH**

Supplementary figure 3A. Western Blot full-length blots.

Supplement: Supplementary file 10 — Supplementary Material 10 [file 12871_2024_2462_MOESM10_ESM.docx]
